# Effect of Crystalline Admixture and Superabsorbent Polymer on Self-Healing and Mechanical Properties of Mortar

**DOI:** 10.3390/ma15176040

**Published:** 2022-09-01

**Authors:** Hexiang Wu, Xi Chen, Yao Liu, Shuangxin Li, Hongfeng Li

**Affiliations:** School of Civil Engineering, Northeast Forestry University, Harbin 150040, China

**Keywords:** crystalline admixture, superabsorbent polymer, self-healing, strength recovery

## Abstract

In this study, the self-healing properties of mortars mixed with a crystalline admixture (CA) and superabsorbent polymer (SAP) were investigated. By conducting uniaxial compressive strength tests on the mortar samples, the effects of the two admixtures and different admixture ratios on the initial compressive strength and strength repair ability at different curing ages of the mortar after pre-cracking were investigated. To verify the results, optical microscopy, scanning electron microscopy, and X-ray diffraction were used for microscopic observation of the cracks and their healing products. The results of this study show that CA, which generates dense substances through chemical reactions, has obvious advantages in the self-healing of microcracks and has a greater effect on the flexural strength of mortar compared with SAP, which can effectively fill wider cracks, reduce the width of cracks through physical expansion, and has a greater impact on the compressive strength of mortar compared with CA. Compared with ordinary mortar, mortar mixed with CA only, and mortar mixed with SAP only, the appropriate amounts of both CA and SAP can effectively combine the advantageous effects of CA and SAP and optimise the self-healing effect of mortar so that its self-healing rate reaches 103%. The self-healing filler, consisting mainly of calcium silicate and calcium carbonate, is generated in cracks and enhances the repair strength of the mortar so that the strength of the mortar reaches 46 MPa.

## 1. Introduction

Concrete is widely used in infrastructure construction because of its advantageous characteristics, such as high strength, low price, and high durability. However, in practice, cement-based materials often deteriorate in performance, even becoming damaged before their expected service life, owing to poor structural design, construction defects, and physical, chemical, or biological erosion caused by various environments. To ensure strength and improve service life, cracks in concrete must be repaired. The conventional solution is to use manual repairs, but the time and economic costs are enormous. The annual direct cost of maintaining concrete roads owing to corrosion in the United States is approximately $4 billion [1]. In Europe, nearly 50% of the construction budget is spent on maintenance and repair of existing structural concrete facilities [2]. Moreover, while the manufacturing cost of concrete ranges from $65 to 80/m^3^, the cost of crack repair and maintenance can reach $147/m^3^ [3]. In addition to the high repair costs, this repair method is also problematic in terms of durability, which is limited to 10–15 years [4]. Additionally, the manual repair method can only address surface cracks, and internal cracks are difficult to repair [5].

With the continuous promotion of concrete in engineering applications, scholars and engineers have gradually advanced their research and understanding of concrete and have found that concrete possesses a certain degree of self-healing properties. However, the self-healing properties of concrete can only repair some minor cracks [6,7,8]. Moreover, the controllability is not strong, and there is a certain randomness [9], which makes it difficult to meet the actual needs of the project. To exploit the self-healing properties of concrete completely, some scholars have added crystalline admixtures (CAs) to improve the self-healing ability. The addition of CA can stimulate the self-healing process and enhance the hydration and recrystallisation processes in the presence of water, including further hydration of unhydrated cement and carbonation of dissolved calcium ions leached from the concrete matrix [10,11,12,13,14,15,16]. CA contains some activating groups that combine with calcium ions in the concrete matrix to form unstable complexes that can form modified calcium silicate with unhydrated cement and water. Reddy and Ravitheja [17] found that adding CA can increase the compressive strength of pre-cracked concrete. They were able to increase the compressive strength by approximately 11.45% compared with the control concrete. This was achieved by the filling of cracks with calcium silicate hydrate and calcite. Chandraiah and Reddy [18] found that CAs play a positive role in the recovery of compressive strength during crack repair, and the addition of crystalline mixtures can effectively reduce the depth of penetration and decrease the permeability coefficient [19]. CA, as a concrete healing admixture, has certain shortcomings, such as the ability to repair cracks of small width only, longer repair times, and a decrease in the initial compressive strength.

Superabsorbent polymers (SAP) are cross-linked polyelectrolytes containing a hydrophilic network structure of covalent bonds capable of absorbing hundreds of times their dry weight in aqueous solutions (or other solutions) [20,21,22]. SAP has efficient internal curing properties and can significantly reduce or even eliminate the self-shrinkage of concrete, improve the performance of concrete, and freely design the shape and size distribution of pores [23]. The addition of SAP to cementitious materials effectively reduces fluid leakage in concrete [24,25]. Mönnig et al. [26] found that SAP can leave a system of entrained air voids in concrete and can improve the freeze–thaw resistance and durability of concrete. In the event of concrete damage, SAP physically fills the cracks by rapidly absorbing water, reducing the size of cracks, and creating favourable conditions for repairing concrete. The research of scholars on SAP in concrete self-healing in recent years is shown in Table 1. SAP has the obvious advantage of physically filling cracks; however, in this process, SAP expands and shrinks, leaving some tiny pores, which affects the compressive strength of the concrete. Some engineering cases and experimental research results have also shown that cracks in concrete cannot completely self-heal when only SAP is added as it inevitably affects the compressive strength of the concrete [27,28]. Currently, there are limited studies on the strength of concrete with added SAP after the self-repair of cracks. In order to make the use of CA and SAP more relevant to engineering practice, the repair rate of the strength of mortar and the apparent conditions of crack repair are investigated in this paper.

In this study, based on the different effects of CA and SAP in the self-healing process of concrete, we analysed the effects of matching the admixture amount while adding CA and SAP simultaneously on the self-healing performance and post-repair strength. We focused on the coupling mechanism of CA and SAP on the self-healing properties of concrete to improve the self-healing performance in terms of strength and impermeability.

## 2. Materials and Methods

Experimental materials were selected from ordinary Portland cement (P. O 42.5R), standard sand (particle size 0.08–0.16 mm, SiO_2_ content >96%), and SAP manufactured by Jinan Huadi Industry and Trade Co., Ltd. SAP (Jinan, China), which is a product of sodium hydroxide and polyacrylic acid neutralization and has a particle diameter of approximately 0.15 mm. CA is a synthetic cementitious material with a particle size of about 0.04 mm, containing mainly reactive chemical materials such as OPC and fine silica. The chemical compositions of the cement and CA are listed in Table 2.

The samples required for all experiments were designed and prepared with a water–cement ratio of 0.5 [32]. The fit ratios of the components for different experimental samples are listed in Table 3. The control samples were named Group O. The samples mixed with CA alone were named Group A. The samples mixed with only SAP were named Group B. The samples mixed with CA and SAP at different dosing ratios were designated as Groups C, D, and E. The amount of SAP is relatively low, which is because if the SAP content is too high its influence on the strength of the mortar is relatively large [33]. For each doping ratio, nine 40 × 40 × 40 mm^3^ and three 40 × 40 × 160 mm^3^ specimens were prepared for different experimental analyses. The materials were mixed in a mixer at low speed for 30 s in a dry state. After adding water, mixing was continued at low speed for 1 min, followed by mixing at high speed for 1.5 min. The finished mortar was poured into the mould and demoulded for 24 h. Finally, the demoulded specimens were cured in a standard maintenance room at 20 ± 2 °C at a relative humidity of more than 95% for 28 days.

A YAW-300H universal testing machine (Jinan Henrui Jin Testing Machine Co., LTD, Jinan, China) was used to test the compressive strength of the square specimens after 28 days of curing. The compression rate was controlled at 2.4 KN/s. The specimens that developed cracks after compression were cured in water at a temperature of 20 ± 2 °C [34]. The specimens were tested again for compressive strength after pre-cracking and curing for 3, 7, and 28 days.

The cracked specimens were wrapped with plastic film and then placed in water at a temperature of 20 ± 2 °C. The recovery of cracks was observed via optical microscopy after 3, 7, and 28 days of curing.

In order to detect the healing products at the cracks, specimens were taken from the cracks of the repaired samples and made into thin slices and powders to observe the microscopic morphology of the healing products at the cracks by scanning electron microscopy (SEM) and X-ray diffraction (XRD), respectively. In this study, samples of the healing products at the cracks were obtained from the control group and specimens containing only CA, only SAP, and both CA and SAP. Moreover, the mechanism of the action of CA and SAP in the self-healing process of concrete was investigated by comparative analysis.

## 3. Results and Discussion

### 3.1. Material Properties

#### 3.1.1. Compressive Strength

**Figure 1:**
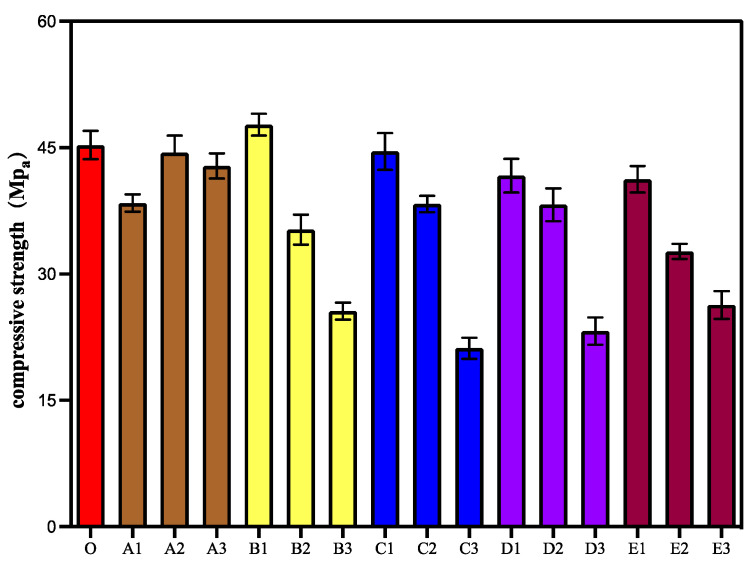
28-day compressive strength of different specimens.

Compressive strength, which is a basic mechanical property of concrete, is a common index used to characterise the performance of concrete. The experimental results of the 28-day compressive strength of different specimens are shown in Figure 1. As shown in the figure, there is a small reduction in the compressive strength of specimens in Group A compared with the control mortar because the addition of CA to the cement results in the consumption of cement and water during the hydration process, according to ACI Committee 212 [35], thereby affecting the hydration of cement to some extent and resulting in reduced mortar strength. ACI 212 reports that silicates of tricalcium are CA-reacted cement-based compounds, and other authors point out the reactive nature of the calcium hydroxide [17]. According to the ACI Committee 212 report, the general process follows Equation (1) [35], in which tricalcium silicates and water react with a crystalline M_X_R_X_ promoter, where M represents some metal ions and R represents some reactive groups. MxRx is primarily an unstable complex containing calcium ions, thus forming modified calcium silicate hydrates and pore blocking precipitates with cement and water. Additionally, the peak compressive strength was obtained when the amount of CA incorporated in the cement was between 0.5% and 2%. The compressive strength of the mortar specimens in Group B decreased with an increase in the SAP admixture content. However, the compressive strength of mortar was higher than that of the control group when the amount of SAP admixture was 0.2%. Thus, peak compressive strength was observed when the amount of SAP admixture in the cement was between 0 and 0.2%. Because SAP absorbs water and expands when there is excess water in the mortar, and when there is a shortage of water inside the mortar, SAP releases the water inside it and promotes the hydration of the cement inside the mortar. [36,37]. However, with the increase in SAP content, microporosity increases in the process of releasing water, thereby reducing the compressive strength of the mortar [33,38]. The compressive strength of Groups C, D, and E showed an overall decreasing trend with the increase in admixture content. When the SAP admixture content was 0.2% (C1, D1, E1), the compressive strength of the mortar was close to that of the control group and exceeded that of Group A. This was because of the release of water from SAP during the reaction of CA. This reduced the adverse effect of CA on the hydration reaction of cement. However, the products of the CA reaction filled the micropores formed by the release of water from SAP. These reaction processes improved the hydration of the cement in Groups C1, D1, and E1 and increased the compressive strength of the mortar. It can be seen that by adding appropriate amounts of CA and SAP at the same time, the advantageous effects of CA and SAP can be combined effectively to improve the compressive strength of mortar. Furthermore, it can be observed from the figure that no peak compressive strength was observed when the CA content was between 0.5% and 2% when a defined amount of SAP was incorporated. This is owing to the stress concentration of micropores formed by the release of water from SAP. Moreover, SAP has a greater effect on the compressive strength of mortar than CA.

(1) $$3\mathrm{CaO}-{\mathrm{SiO}}_{2}+M_{x}R_{x}+H_{2}O\to {\mathrm{Ca}}_{x}{\mathrm{Si}}_{x}O_{x}R-{\left(H_{2}O\right)}_{x}+M_{x}{\mathrm{CaR}}_{x}-{\left(H_{2}O\right)}_{x}$$

Calcium silicate + crystalline enhancer + water → modified calcium silicate hydrate + pore blocking precipitate

#### 3.1.2. Flexural Strength

**Figure 2:**
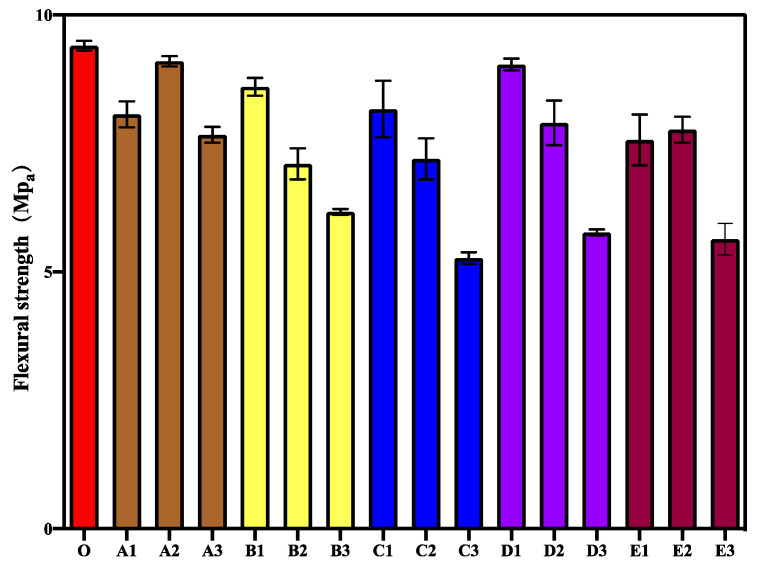
28-day flexural strength of different specimens.

Considering the different stress positions and states, the effect of mixing CA and SAP on the flexural strength of mortar was further investigated in this study. The experimental results of the 28-day flexural strength tests of different specimens are shown in Figure 2. As shown in the figure, the trend of the flexural strength of different specimens is basically the same as that of the compressive strength. However, the main difference is that when both CA and SAP are added, the peak flexural strength occurs when the CA content ranges from 0.5% to 2% depending on the determined SAP admixture content. Improved flexural strength was observed in mortar containing 0.2% SAP and 1% CA. This is because CA affects the hydration reaction of cement and reduces the joint strength of mortar. Moreover, CA has a greater effect on the flexural strength of mortar than SAP.

#### 3.1.3. Repair Strength

**Figure 3:**
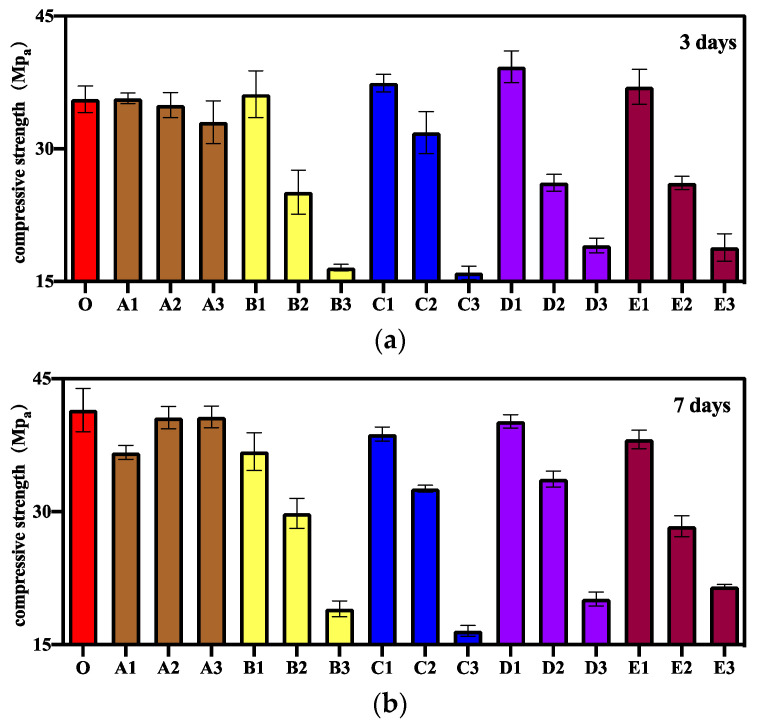

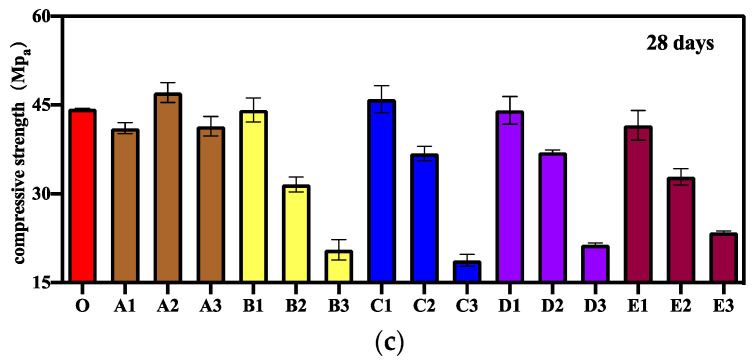
Compressive strength of different specimens after curing in water: (**a**) 3 days, (**b**) 7 days, (**c**) 28 days.

**Figure 4:**
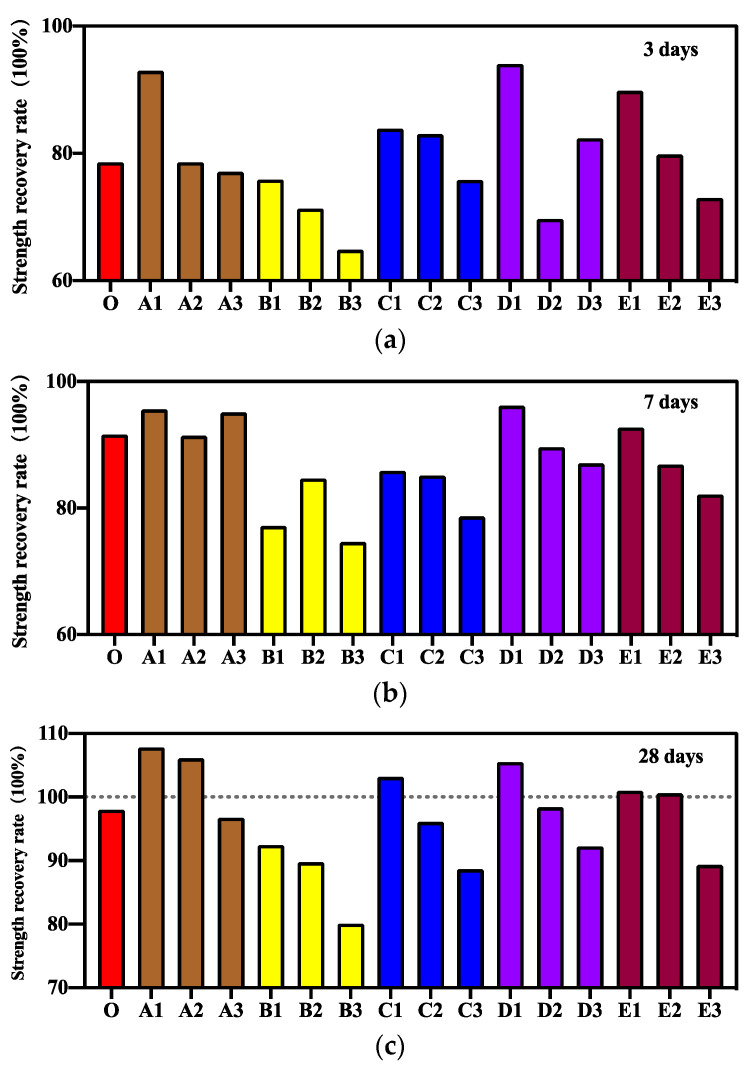
Strength repair rate of different specimens after curing in water: (**a**) 3 days, (**b**) 7 days, (**c**) 28 days.

To reveal the effect of CA and SAP at different ratios on the self-healing performance of concrete, mortars with different curing ages after pre-cracking were subjected to compression experiments to measure the repair strength and strength repair rate. The experimental results of compressive strength and the calculated results of strength repair rate of different samples at different curing ages are shown in Figure 3 and Figure 4, respectively. As shown in Figure 3a and Figure 4a, in the early healing stage after 3 d of pre-cracking curing, the compressive strength of mortar was high when CA content was low, and peak compressive strength was not observed when CA content was between 0.5% and 2%. This is because as the amount of CA increases, it reacts with more cement and has a negative impact on the mortar strength. The mortar mixed with 0.2% SAP also achieved higher strength than the control group in the early healing stage, but the strength repair rate of all specimens in Group B mixed with only SAP was lower because SAP fills the cracks by physical expansion during the crack repair process [20,39,40] and has little effect on strength repair. When CA and SAP are mixed with the mortar simultaneously, the specimens mixed with 1% CA and 0.2% SAP had the highest strength and the highest strength repair rate in the early healing stage. This is because in the self-healing process of mortar, SAP reduces the width of cracks by expansion, enabling CA to have a better repairing effect on tiny cracks [32] and enhancing the strength of the mortar. The addition of CA and SAP at an appropriate ratio can improve the self-healing effect of mortar [30].

The repair strength and strength repair rates of different samples after 7 d of pre-cracking care are shown in Figure 3b and Figure 4b. As shown in the figure, the overall highest repair strength and strength repair rate were observed in Group A specimens because the role of CA in crack repair gradually became obvious in the middle of the healing stage and became the main factor for compressive strength repair. The overall compressive strength and strength repair rates of Group B specimens were lower because SAP filled the cracks by physical expansion and had a more obvious advantage in the early healing stage where large cracks existed [41]. However, the effect was not obvious in the middle healing stage, where only micro cracks existed. When CA and SAP were mixed in the mortar simultaneously, 0.2% SAP content had a more obvious advantage in strength repair compared with other specimen doses.

The repair strength and strength repair rate of different samples after 28 d of pre-cracking care are shown in Figure 3c and Figure 4c. As shown in the figure, the strength repair rates of specimens in Groups A1 and A2 reached 107.8% and 106.1%, respectively, and the repair strength of specimens in Group A2 exceeded the initial compressive strength of the control group. This is because CA increases the mortar density during the reaction and obtains the product for repairing cracks. This finding is similar to some existing research results, which state that a certain amount of CA can improve the strength repair rate by more than 100% [42]. The difference is that the repair strength and strength repair rate of the A3 group specimens are lower in the final healing stage because of mixing excessive CA, which does not react, thereby reducing the strength of mortar during the crack repair process. The Group B specimens did not show satisfactory results in terms of compressive strength repair. In the samples mixed with CA and SAP simultaneously, by controlling the SAP content at 0.2%, we were able to obtain an improved repair strength and strength repair rate. The specimen in Group C1 mixed with 0.5% CA showed the maximum repair strength in the final healing stage at 28 days. The low strength repair rate was owing to the higher initial compressive strength.

Based on the above analysis, the initial compressive and flexural strengths of different samples, and the strength repair ability shown at different curing ages after pre-cracking, it is apparent that the condition of the crack repair varies with the timing of maintenance and is dependent on the dopants used. Most possibly, this is the result of the production rate of the mortar healing product during the various reactions. The optimal amounts of 0.5% CA and 0.2% SAP can effectively combine the physical expansion of SAP to reduce the crack width and the chemical reaction products of CA to fill the tiny cracks. This not only improves the healing effect of the mortar but also enhances its repair strength and thereby improving its mechanical properties.

### 3.2. Healing Products

#### 3.2.1. Crack Healing Pattern

**Figure 5:**
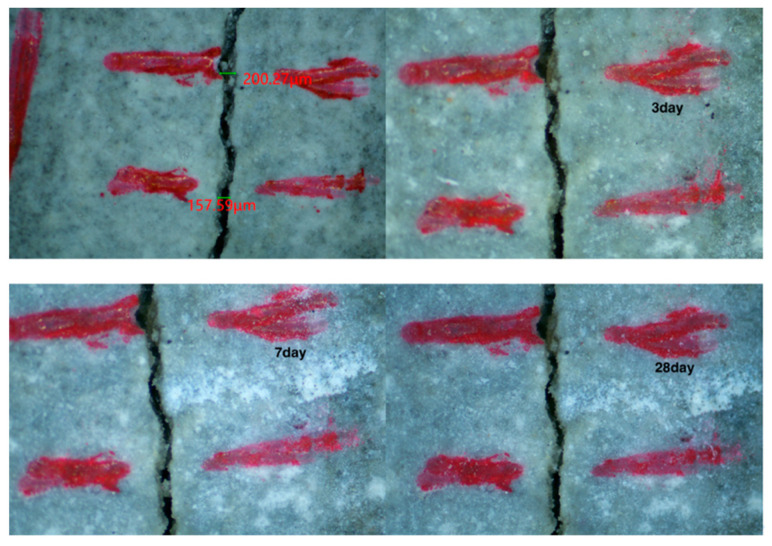
Self-healing of the control mortar at 3, 7 and 28 days.

**Figure 6:**
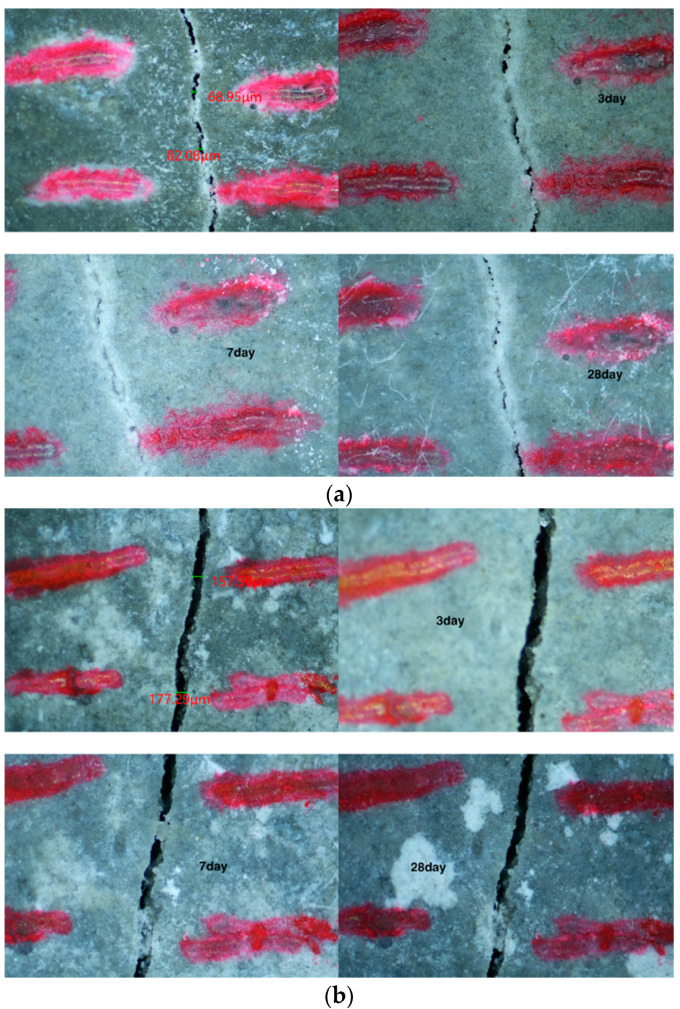
Self-healing of mortar mixed with CA only at 3, 7 and 28 days: (**a**) small cracks, (**b**) large cracks.

**Figure 7:**
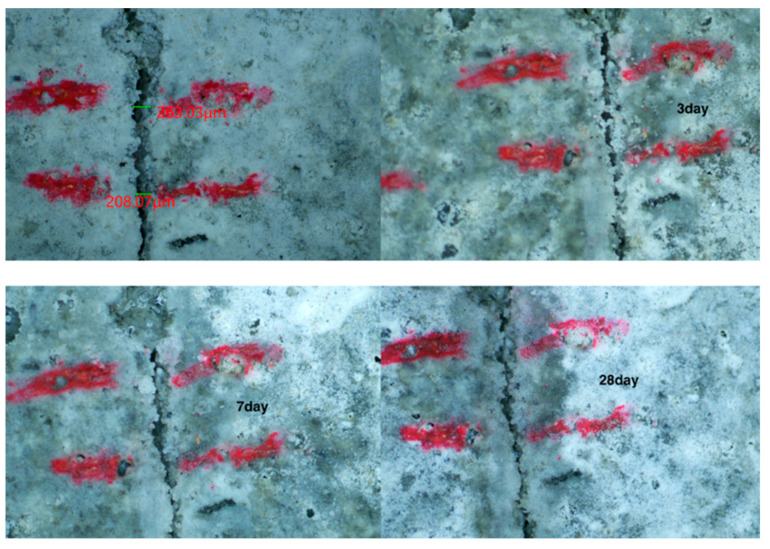
Self-healing of mortar mixed with SAP only at 3, 7 and 28 days.

**Figure 8:**
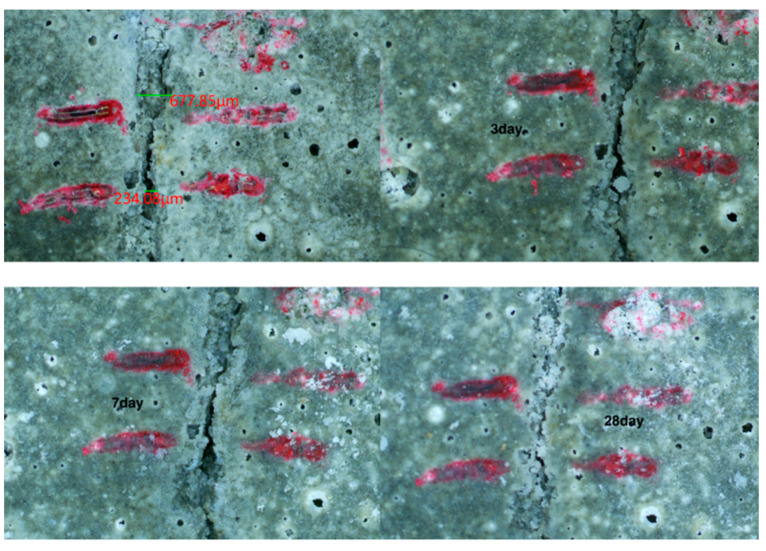
Self-healing of mortar mixed with CA and SAP simultaneously at 3, 7 and 28 days.

The self-healing performance of mortar can be judged visually by observing the healing state of cracks on the specimen surface. The recovery of cracks on the surface of each specimen group observed via a stereo microscope is shown in Figure 5, Figure 6, Figure 7 and Figure 8. As shown in Figure 5, the penetration cracks in the control specimens showed very limited crack repair after 3, 7, and 28 d of in-water curing. This is because ordinary mortar can only repair cracks by further hydration of the internal unhydrated cement. This process has a limited effect and can only repair very small cracks. As shown in Figure 6, penetration cracks in specimens mixed with only CA were repaired to some extent by maintenance in water for 3, 7, and 28 days. The repair of cracks with tiny widths was more satisfactory. As shown in Figure 6a, when the crack is about 70 microns, the crack is well repaired. However, the repair of cracks with larger widths was poor, as shown in Figure 6b when the crack is 150 microns, it is difficult for the crack to be repaired. This indicates that the crystalline dopant fills the cracks through the chemical reaction to generate products, and the effect is obvious for cracks with small widths. However, this effect on cracks with larger widths is limited. As shown in Figure 7, in specimens containing only SAP, the penetration cracks were repaired to some extent by maintenance in water for 3, 7, and 28 days. Compared with the specimens mixed with only CA, it can be observed that the effect of SAP is more obvious for cracks with a width of more than 200 microns. More than half of the crack width can be repaired by 28 days of maintenance in water because SAP fills the cracks by absorbing water and expanding, thereby reducing the width of the cracks and resulting in a better healing effect. As shown in Figure 8, for specimens mixed with CA and SAP, the penetration cracks were repaired by curing in water for 3, 7, and 28 days. Compared with the specimens mixed with only CA or SAP, it can be observed that the specimens mixed with CA and SAP showed excellent self-healing performance in the full cycle of self-healing for the same large width cracks, with the cracks almost filled after 28 days maintenance in water. This is a combination of the advantages of CA and SAP in the self-healing process of mortar: SAP absorbs water and expands to fill the wide cracks, whereas CA reacts to generate products that fill the tiny cracks. Thus, the mortar is denser, which improves its self-healing effect.

#### 3.2.2. Microscopic Analysis Results

**Figure 9:**
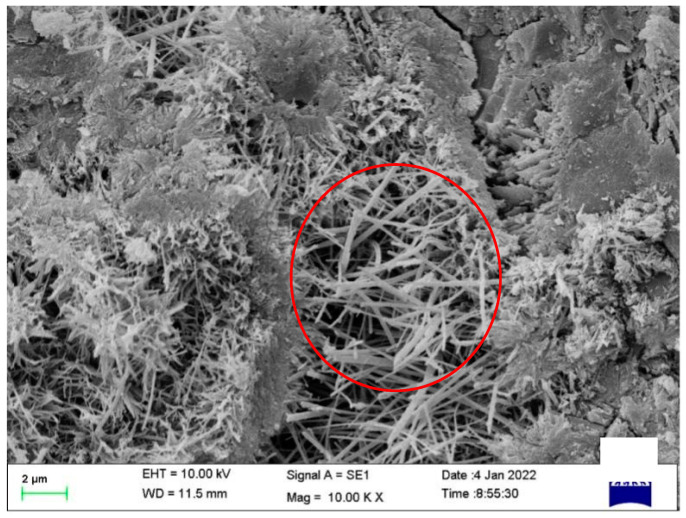
The morphology of the healing products of the S1 specimen.

**Figure 10:**
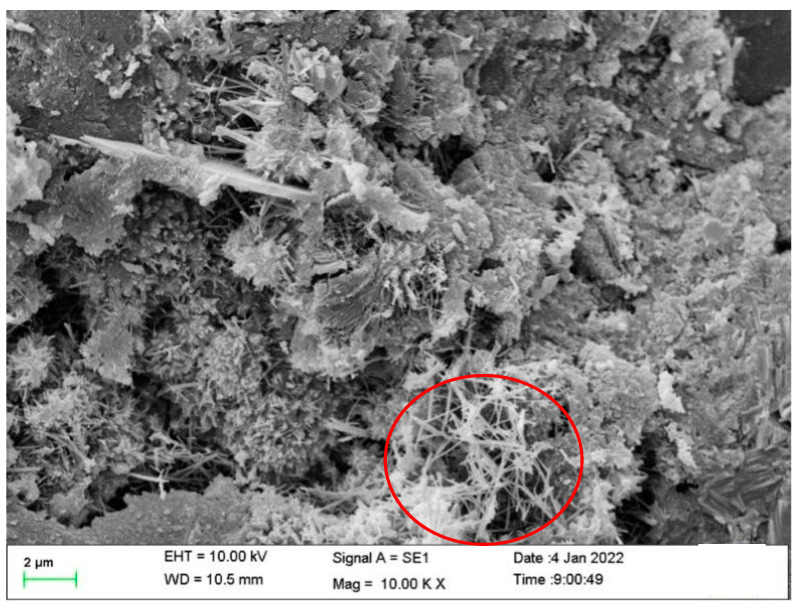
The morphology of the healing products of the S2 specimen.

**Figure 11:**
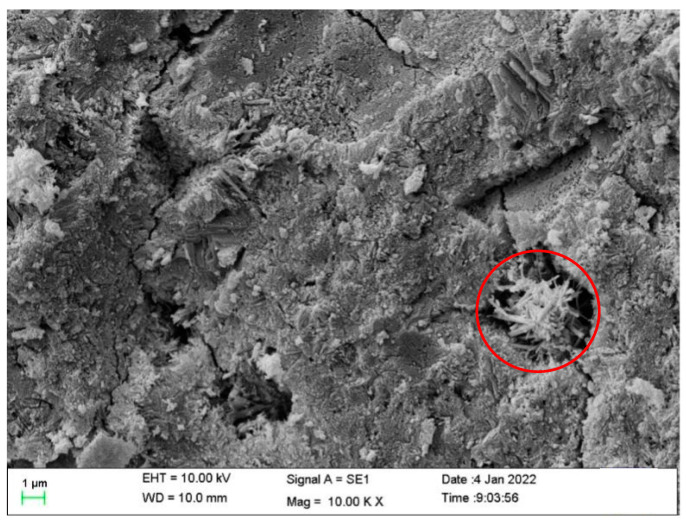
Morphology of healing products of the S3 specimens.

To further investigate the mechanism of property changes after healing, the morphologies of the healing products were observed using SEM, and the chemical compositions of the healing products were observed using XRD. This information was used to determine the type of healing product. The morphologies of the mortar healing products obtained by adding CA only (S1), SAP only (S2), and both CA and SAP (S3) are shown in Figure 9, Figure 10 and Figure 11 (red circle). As shown in the figure, the healing products are needle-like crystals, which agrees with the findings of Wang et al. [43]. Additionally, it can be observed from the figure that the mortar healing products obtained by mixing with only CA were denser, the mortar healing products obtained by mixing with only SAP were agglomerated into spherical shapes and sparse, and the mortar healing products obtained by mixing with CA and SAP were also agglomerated into spherical shapes, but denser. Based on the needle-like crystalline morphology, the healing product is judged to be calcium silicate. By comparing the spherical crystals shown in Figure 10 and Figure 11, it is judged that the healing products are preferentially generated on the surface of SAP, and the difference in the degree of denseness is the effect of the chemical reaction products of CA. This is consistent with the dense crystals generated by CA, as shown in Figure 9, reflecting the effect of coupling CA and SAP with complementary advantages.

**Figure 12:**
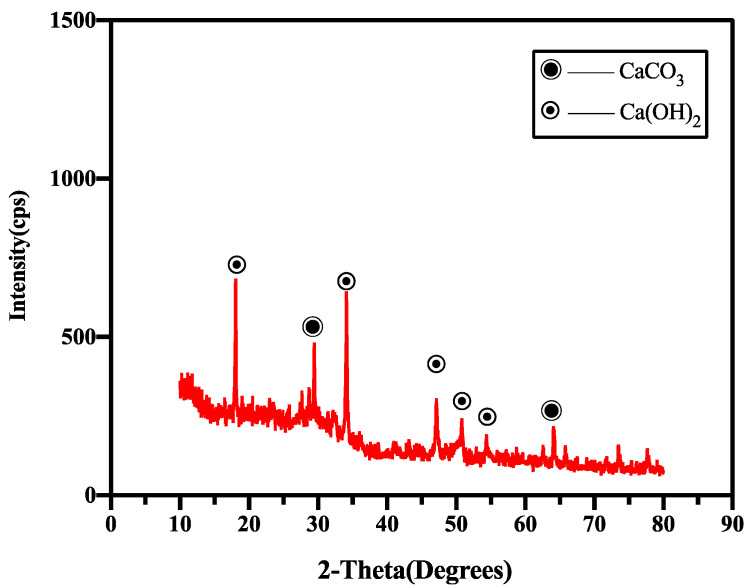
XRD spectra of the products of group X1.

**Figure 13:**
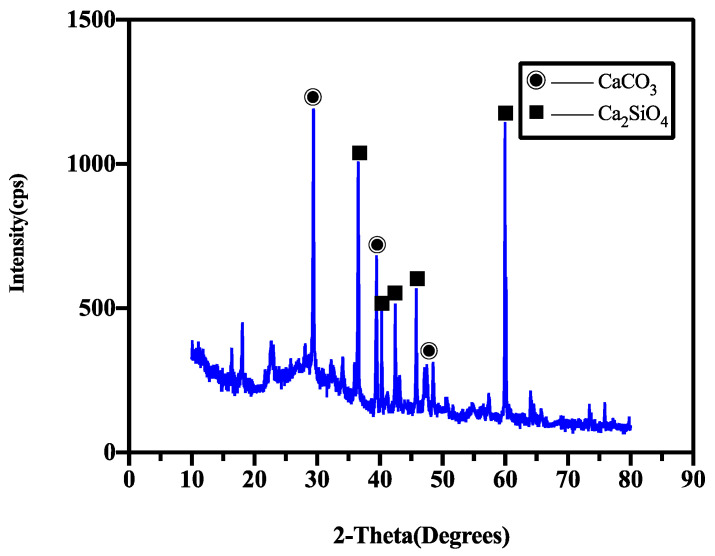
XRD spectra of the products of group X2.

**Figure 14:**
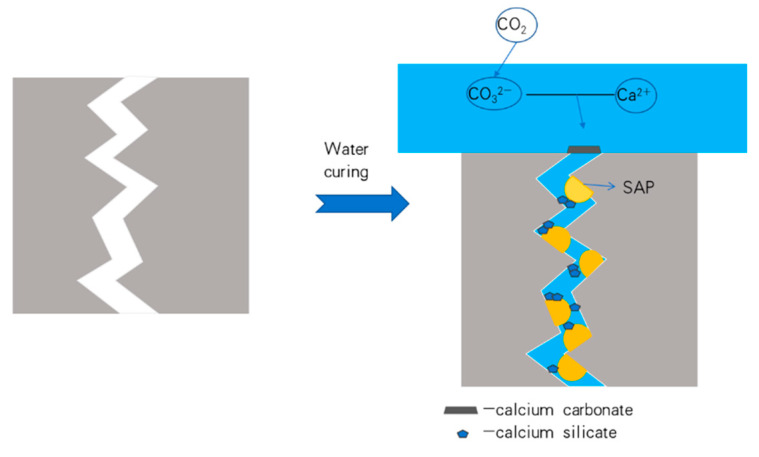
Schematic diagram of CA and SAP coupling role.

The XRD analysis results of the healing products of the control group (X1) and the specimens mixed with CA and SAP simultaneously (X2) are shown in Figure 12 and Figure 13, respectively. As shown in the figure, the control healing product contained mainly calcium hydroxide and calcium carbonate. The healing products of the CA and SAP groups were calcium carbonate and calcium silicate. It is evident that compared with the control group, the CA and SAP groups have no peak in the interval represented by calcium hydroxide in the XRD analysis graph, whereas the interval represented by calcium silicate has a peak, and the peak representing calcium carbonate has a shift to the left in the 25–30 interval. In addition, SAP does not produce a chemical reaction in mortar, indicating that the crystalline admixture consumes calcium hydroxide and produces calcium silicate during the chemical reaction, and the combination of CA and SAP forms a denser calcium carbonate. In addition to the SEM and XRD analysis results, it can be observed that there is a large amount of silicate ions, which fill the cracks by combining with free calcium ions to form calcium silicate inside the mortar. At the surface of the cracks, the combination of water and carbon dioxide in the air produces large amounts of carbonate ions that can combine with free calcium ions to form calcium carbonate products. Therefore, the self-healing product in the mortar cracks is primarily calcium silicate, whereas the self-healing products on the crack surface are mainly calcium silicate and calcium carbonate. The working principle of the coupling action between CA and SAP is shown in Figure 14. SAP reduces the internal crack width by expansion to provide better conditions for the CA reaction, which produces calcium silicate to fill the internal cracks. Meanwhile, carbon dioxide in the air and water form carbonate ions with calcium ions in the specimen to form calcium carbonate that covers the crack surface.

## 4. Conclusions

In this study, the effects of two dopants with different mechanisms of action and dosages on the mechanical and self-healing properties of mortar were investigated by adding CA and SAP in different amounts independently and together. The specimens were cured by pre-cracking and then placed in water for 3, 7, and 28 days. The effects of CA and SAP at different ages and the recovery of mechanical properties of the mortar were investigated. Additionally, microscopic observation and identification of the repair of mortar cracks and the generated filling products were performed using optical microscopy, SEM, and XRD. The conclusions drawn from the experiments are as follows:

1. SAP has a greater effect on the compressive strength of mortar compared with CA, whereas CA has a greater effect on the flexural strength of mortar compared with SAP. Mixing CA and SAP simultaneously and controlling the dosing ratio can effectively combine the advantageous effects of CA and SAP to improve the mortar strength.
2. SAP can effectively reduce the width of cracks through physical expansion, whereas CA can fill microcracks through chemical reactions to produce a dense material. Compared with the control mortar, mortar mixed with only CA, and mortar mixed with only SAP, mortar mixed with appropriate amounts of CA (0.5%) and SAP (0.2%) can optimise the self-healing effect and enhance the repair strength so that the strength of mortar after repair reaches 46 MPa and the strength repair rate reaches 103%.
3. It was observed by optical microscopy that CA has obvious advantages in the self-healing of microcracks. However, this effect is limited to wider cracks. SAP can fill wider cracks effectively, but not completely. The self-healing performance of mortar can be improved by combining the advantages of CA and SAP.
4. From the SEM and XRD analyses, it can be determined that the self-healing filler generated in the cracks is primarily calcium silicate, whereas the healing products generated on the crack surface are primarily calcium silicate and calcium carbonate in the mortar mixed with CA and SAP.

## Figures and Tables

**Table 1:** Research about SAP.

Reference No.	Test	Result
[29]	Water flow test	The in-house made SAPs with a particle size between 400 and 600 μm performed the best with regard to crack closure.
[30]	Water flow test	Larger SAP sizes improved the self-sealing capability.
[31]	X-ray tomography	The mix with 2.2% superplasticizer at 40% damage level had a full recovery from cracks (i.e., healing). This is due to forces from the SAPs’ expansion which closes cracks.
[32]	rapid permeability test (RPT)	The crack healing efficiency of a SAP-added specimen was quite satisfactory, but the complete repair was hard to achieve.

**Table 2:** Main chemical composition of cement and CA.

Oxide Content (%wt)	OPC	CA
CaO	64.84	57.50
${\mathrm{SiO}}_{2}$	20.14	15.32
${\mathrm{Al}}_{2}O_{3}$	4.94	5.18
${\mathrm{Fe}}_{2}O_{3}$	3.83	2.48
${\mathrm{Na}}_{2}O$	0.25	1.83
$K_{2}O$	0.88	0.80
${\mathrm{SO}}_{3}$	3.02	2.90
MgO	1.34	13.20

**Table 3:** Dosage of different mortar specimen components.

Mortar		Sand	Cement	CA	CA/C	SAP	SAP/C	Water
		(g)	(g)	(g)	(%)	(g)	(%)	(g)
O		1800	900					450
A	A1	1800	895.5	4.5	0.5			450
	A2	1800	891	9	1			450
	A3	1800	882	18	2			450
B	B1	1800	900			1.8	0.2	450
	B2	1800	900			4.5	0.5	450
	B3	1800	900			9	1	450
C	C1	1800	895.5	4.5	0.5	1.8	0.2	450
	C2	1800	895.5	4.5	0.5	4.5	0.5	450
	C3	1800	895.5	4.5	0.5	9	1	450
D	D1	1800	891	9	1	1.8	0.2	450
	D2	1800	891	9	1	4.5	0.5	450
	D3	1800	891	9	1	9	1	450
E	E1	1800	882	18	2	1.8	0.2	450
	E2	1800	882	18	2	4.5	0.5	450
	E3	1800	882	18	2	9	1	450

## Data Availability

No new data were created or analyzed in this study. Data sharing is not applicable to this article.
